# TELE-SAVVY@HOME: CONVERTING TO A COMPLETELY ASYNCHRONOUS DELIVERY APPROACH

**DOI:** 10.1093/geroni/igac059.1702

**Published:** 2022-12-20

**Authors:** Fayron Epps

**Affiliations:** Emory University, Fairburn, Georgia, United States

## Abstract

It is well established that family caregiving is taxing and stressful. Group-based psychoeducational programs such as the Tele-Savvy program demonstrated that the acquisition of skills, knowledge, and caregiving mastery can ameliorate caregiving stress – and enhance PLWD quality of life. Many factors, however, preclude caregivers’ attendance in the synchronous portions of Tele-Savvy limiting the program’s scalability. To make Tele-Savvy more accessible and available to a growing number of dementia family caregivers, we designed a fully asynchronous version of the Tele-Savvy program, Tele-Savvy@Home. To accomplish this, we consulted facilitators and caregivers who participated in Tele-Savvy. We also reviewed the existing curriculum, re-wrote portions, reordered modules, and created new videos to cover synchronous content. In addition, we partnered with education design specialists to create interactive and reflection exercises to maintain a psychoeducation orientation and strengthen caregivers’ self-efficacy. This strategy may help investigators in their augmentation of the delivery strategies of established interventions.

